# Genetic analysis of heterogeneous subsets of circulating tumour cells from high grade serous ovarian carcinoma patients

**DOI:** 10.1038/s41598-023-29416-z

**Published:** 2023-02-13

**Authors:** Du-Bois Asante, Ganendra R. K. A. Mohan, Emmanuel Acheampong, Melanie Ziman, Leslie Calapre, Tarek M. Meniawy, Elin S. Gray, Aaron B. Beasley

**Affiliations:** 1 Centre for Precision Health, Edith Cowan University, Joondalup, WA 6027 Australia; 2 School of Medical and Health Sciences, Edith Cowan University, Joondalup, Perth, WA 6027 Australia; 3 School of Medicine, University of Notre Dame, Fremantle, WA 6160 Australia; 4 School of Biomedical Science, University of Western Australia, Crawley, WA 6009 Australia; 5 School of Medicine, University of Western Australia, Crawley, WA 6009 Australia; 6 Department of Medical Oncology, Sir Charles Gairdner Hospital, Nedlands, WA 6009 Australia

**Keywords:** Ovarian cancer, Prognostic markers

## Abstract

Circulating tumour cells (CTCs) are heterogenous and contain genetic information from the tumour of origin. They bear specific intra- and extra-cellular protein markers aiding in their detection. However, since these markers may be shared with other rare cells in the blood, only genetic testing can confirm their malignancy. Herein, we analyse different CTC subsets using single cell whole genome DNA sequencing to validate their malignant origin. We randomly selected putative CTCs identified by immunostaining that were isolated from 4 patients with high grade serous ovarian cancer (HGSOC) and one with benign cystadenoma. We specifically targeted CTCs positive for epithelial (CK/EpCAM^pos^), mesenchymal (vimentin^pos^), and pseudoendothelial (CK/EpCAM^pos^ plus CD31^pos^) markers. We isolated these cells and performed whole genome amplification (WGA) and low-pass whole-genome sequencing (LP-WGS) for analysis of copy number alterations (CNA). Of the CK/EpCAM^pos^ cells analysed from the HGSOC patients, 2 of 3 cells showed diverse chromosomal CNAs. However, the 4 pseudoendothelial cells (CK/EpCAM^pos^ plus CD31^pos^) observed in the HGSOC cases did not carry any CNA. Lastly, two of the clusters of vimentin positive cells sequenced from those found in the benign cystadenoma case had CNA. Despite the low number of cells analysed, our results underscore the importance of genetic analysis of putative CTCs to confirm their neoplastic origin. In particular, it highlights the presence of a population of CK/EpCAM^pos^ cells that are not tumour cells in patients with HGSOC, which otherwise would be counted as CTCs.

## Introduction

Circulating tumour cells (CTCs) are rare neoplastic cells found in the circulatory system, which are shed from primary, metastatic or recurrent tumours^1,2^. They can be phenotypically and genotypically heterogeneous; with each cell potentially acting as a precursor of the tumour of origin^3^.

So called “classical” CTCs from carcinomas carry epithelial traits that can be detected by the expression of EpCAM and cytokeratins (CK). However, some CTCs may acquire mesenchymal traits, expressing markers such as vimentin, while failing to express epithelial markers^4^. In fact, epithelial to mesenchymal transition (EMT) is a key process driving cancer cell dissemination and metastases^5^. Similarly, expression of vascular endothelial-cadherin (VE-cadherin) on a subpopulation CTCs indicates the acquisition of endothelial-like properties by tumour cells^6^. It has become apparent that certain CTCs subpopulation are indicative of a more aggressive disease and potentially reveal new therapeutic targets^7,8^.

The advent of new technologies has been crucial for advancing the application of CTC as a liquid biopsy for precision medicine^9,10^. Though detection via immunocytochemistry has been the traditional way of identifying CTCs in patients’ blood, the inclusion of molecular analysis in the past two decades has enhanced validation and further molecular characterisation of detected CTCs^11–13^. Some of these technologies include molecular analysis of identified CTCs by means of gene expression^14^ and chromosomal copy number alterations (CNA)^15,16^. These methods do not only confirm the neoplastic origin of these cells, but also aid in the prediction of disease progression and selection of treatment strategies^17^.

In our recent study, we identified heterogeneous CTCs in ovarian cancer (OC) patients using a multi-marker immunostaining method for the detection of both epithelial and mesenchymal CTCs^18^. Mesenchymal CTCs were identified as vimentin positive cells. But due to the expression of vimentin in circulating endothelial cells (CECs), CD31 was used as exclusion marker, similar to other studies^19,20^. We previously noted putative CTCs expressing CK/EpCAM as well as CD31 positive^18^, presenting as pseudoendothelial phenotype. Recently putative tumorigenic CECs with aneuploidy have been reported from OC^21^ and lung cancer patients ^22^. Thus, we aimed to evaluate the neoplastic origin of the putative CTCs identified by carrying out genetic analysis for CNAs. Notably, high serous ovarian cancer (HGSOC) is marked by chromosome instability and high burden of CNAs^23^, which underpin our approach.

## Results

We randomly selected 20 putative CTCs from a total of 5 patients. Four patients had HGSOC and were found to have 2–49 CTCs as detected in our previous analysis^18^. In addition, a benign case histologically confirmed to be serous cystadenoma, was included as numerous vimentin positive CTC-like cells were present in the patient’s enriched samples.

**Figure 1:**
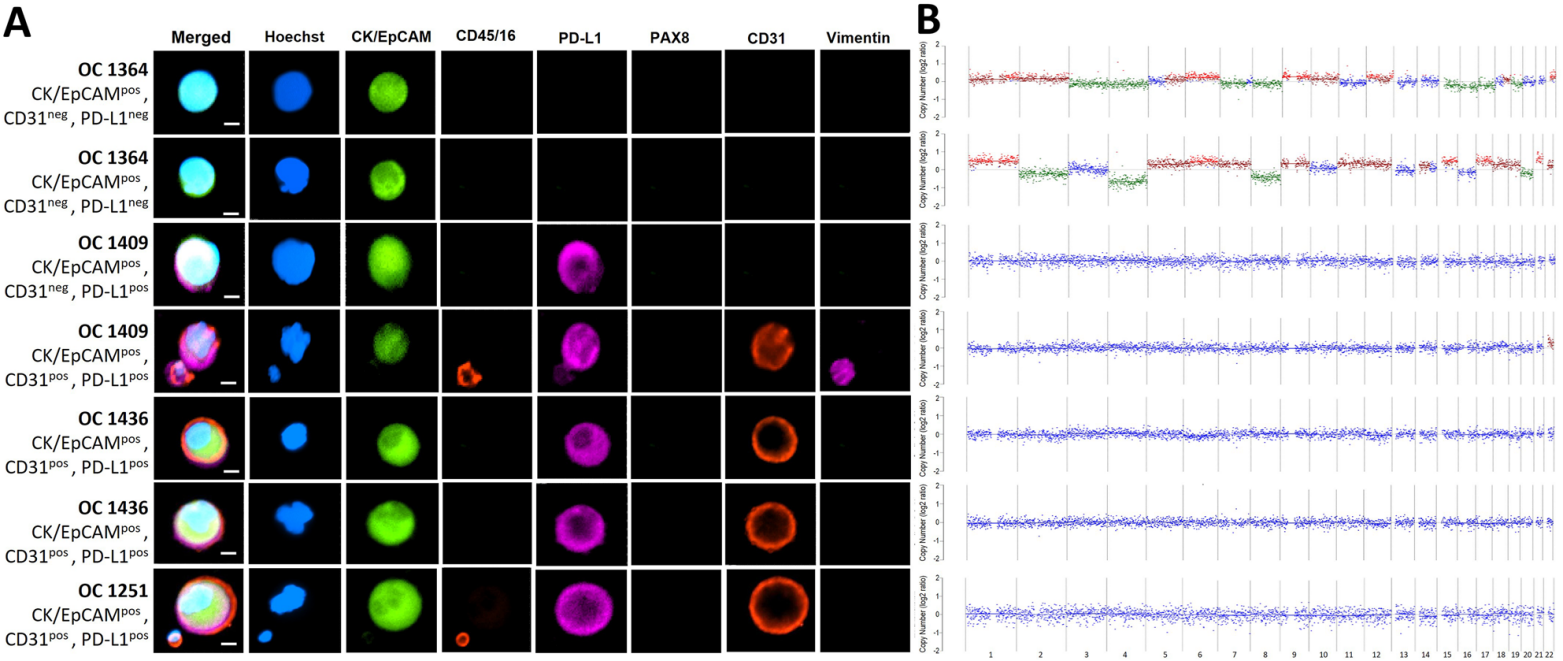
Representative fluorescence images of CTCs and their corresponding CNV profiles. Scale bar (bottom right) represents 10 μm. (**A**) Cells were stained with antibodies for cytokeratin and EpCAM (FITC, green), CD45/CD16 (PE, red), PD-L1 (AF647, cyan) and nuclei staining in blue (Hoechst), in the first panel. It was followed by fluorescence quenching and re-staining with antibodies for PAX8 (FITC, green), CD31 (PE, red) and vimentin (AF647, cyan)^18^. (**B**) CNA profiles obtained from low-pass whole-genome sequencing are shown to the right. Blue dots indicate neutral CNA, red indicate gains, and green indicate losses.

For the HGSOC cases, we selected 15 Hoechst^pos^/CK^pos^/EpCAM^pos^ and CD16/45^neg^ cells. These cells were morphologically distinct with larger and more spherical nuclei compared to the nuclei of WBCs. A proportion of the CK^pos^/EpCAM^pos^ cells were positive for the vascular endothelial marker CD31 and PD-L1 (Fig. 1), which we defined as pseudoendothelial cells. These cells were very rare and only observed in 3 of the 16 patients analysed in our original study^18^.

Of the 15 putative CTCs that were picked from the HGSOC cases for WGA profiling, seven (47%) passed QC (Table 1). Of the three CTCs that were exclusively CK/EpCAM^pos^ (epithelial CTCs), two from patient OC1364 had CNA, as expected from classical CTCs (Fig. 1). These 2 CTCs showed diverse chromosomal CNA patterns despite being derived from the same patient. In contrast, the analysis of the 4 pseudoendothelial cells that were CK/EpCAM^pos^/PD-L1^pos^/CD31^pos^, isolated from patients OC1409, OC1436 and OC1458, showed no chromosomal CNA (Fig. 1), which may suggest that they were not neoplastic cells despite expression of CK/EpCAM.

**Table 1:** Selected cell phenotype and CNV detection.

Cell Phenotype	Markers	# Cells picked	# Cells that passed QC	Cells with CNA (%)	Tumour type
Epithelial	CK/EpCAM^pos^	6	3	2 (66.7%)	HGSOC
Pseudo-endothelial	CK/EpCAM^pos^, PD-L1^pos^ and CD31^pos^	9	4	0	HGSOC
Mesenchymal	Vimentin^pos^	5	2	2 (100%)	Serous cystadenoma

**Figure 2:**
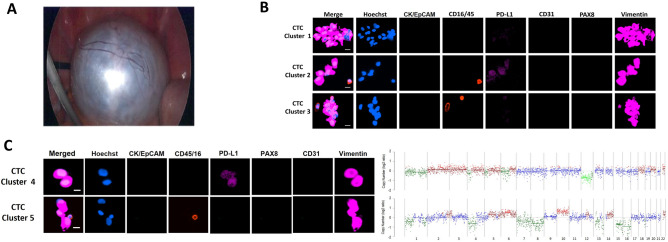
Representative fluorescence images of CTCs and their corresponding CNV profiles from the case with a benign tumour. Scale bar (bottom right) represents 10 μm. (**A**) T Photomicrograph of the tumour from the benign case, which was histologically diagnosed to be serous cystadenoma. Isolated cells (**B** and **C**) from blood were stained with antibodies for cytokeratin and EpCAM and cytokeratin (FITC, green), CD45/CD16 (PE, red), PDL1 (AF647, cyan) and nuclei staining in blue (Hoechst), in the first panel. It was followed by fluorescence quenching and re-staining with antibodies for PAX8 (FITC, green), CD31 (PE, red) and vimentin (AF647, cyan). Detected cells were mainly vimentin positive clusters. (**C**) CNA profiles obtained from low-pass whole-genome sequencing are shown at the far right from randomly selected clusters. Blue dots indicate neutral CNA, red indicate gains, and green indicate losses.

Analysis of CTCs from the patient with benign cystadenoma revealed 9 clusters, with approximately 2 to16 cells per cluster of vimentin expressing cells (Fig. 2). Initially, the benign case was suspected to be malignant as it measured 30 cm by CT scan, though the CA-125 level was only 3 KU/L, which was well below the threshold for positivity. These cells were vimentin^pos^, CK/EpCAM^neg^, CD31^neg^, and CD16^neg^/45^neg^. Only cluster 2 and 4 (Fig. 2B,C), showed weak to moderate expression of PD-L1 respectively on the cells. Genetic analysis of clusters 4 and 5 indicated that these cells carry CNAs suggesting a neoplastic origin despite the presumed benign nature of the lesion from which they were derived (Fig. 2C).

## Discussion

We performed single-cell genomic analysis on three distinct subsets of putative CTCs: epithelial-CK/EpCAM expressing cells, mesenchymal-vimentin expressing cells and pseudo-endothelial-CK/EpCAM plus CD31 expressing cells. Notably, we identified chromosomal CNAs in epithelial and mesenchymal cells suggesting that these were indeed CTCs. There were no CNAs detected in the pseudoendothelial cells analysed.

CNA is a hallmark of serous ovarian cancer tumours^24^, particularly in HGSOCs^23^. The presence of CNAs have been recently reported in CTCs isolated from HGSOC patients^25^. In the study by Salmon et al.^25^, chromosomal CNAs were detected in 67% of the CK/EpCAM positive CTCs analysed, demonstrating evidence of acceptability of these two conventional markers in CTC detection from malignant tumours, from amongst non-tumourigenic CK/EpCAM positive epithelial cells in blood (CellSearch criteria for CTC detection)^26^. Nevertheless, the absence of CNA in 23% of CK/EpCAM CTCs in their study is in line with our finding that a proportion of CK/EpCAM cells are not neoplastic.

The distribution of the chromosomal CNA patterns found on the CK/EpCAM CTCs in our study were similar to the ones from HGSOC tumours reported by TCGA^23^ and CTCs from HGSOC patients^25^. The remarkable difference in chromosomal loses and gains despite being detected in the same patient may be explained by the high chromosomal instability in HGSOC patients and the great genomic heterogeneity of the disease^23,27^.

All pseudoendothelial cells, which were CD31^pos^ in addition to CK/EpCAM^pos^ and expressed PD-L1, had no CNA suggesting that these cells are not neoplastic, and that they may be of vascular endothelial origin. However, this must be regarded as hypothesis generating observation, as we only analysed a limited number of cells from a few patients. Nevertheless, this observation is important as most CTC quantification panels do not include CD31 and these cells would have been considered CTCs due to the expression of CK and EpCAM^18^.

Though there are other markers used for CEC identification such as CD34 and CD146^28^, we chose CD31 because it has shown a clear membrane localization^18^, and it is one of the commonly used markers for the identification of CECs^29,30^. Our findings underscore the need to include endothelial markers for example CD31, as an additional negative selection marker in OC CTC analysis to help reduce false positivity. This will significantly improve the accuracy of quantification of identified CTCs.

Most studies on CTCs have not noted this contamination, as they did not include CEC markers. CECs have been detected in the blood of healthy donors^28^ and elevated cancer patients^31^. In recent years, CECs expressing endothelial markers CD105 and/or CD146 (CellSearch), have been associated with poor patient outcome in metastatic breast^32^ and colorectal cancer^33^. However, only a few studies have reported CECs being detected in OC CTC studies^21,34^. Thus, it would be important to combine CTC and CECs analyses for prognostication in future OC studies.

Moreover, CECs that were PDL-1 positive have been reported to be associated with poor clinical outcome in non-small lung cancer patients^22^. Since, anti-angiogenic therapy, bevacizumab, has been approved in the frontline, platinum-sensitive recurrent and platinum-resistant recurrent settings for epithelial OCs^35^, CD31 expression of circulating vascular cells or levels of serum VEGF in association with response to bevacizumab, will be worth exploiting in OC patients^36^. More applicable to clinical settings, the evaluation of CK/EpCAM^pos^, PD-L1^pos^ and CD31^pos^ cells in patients treated with combined immune- and VEGF inhibitor therapies, may aid in the determination of their prognostic and predictive significance in future studies.

The observed results also demonstrated that the two vimentin^pos^ clusters in the benign case had chromosomal CNA. Though not expected, this is not surprising as CTCs have been reported to be detected from benign colon diseases^37^ and individuals with benign ovarian tumours^38,39^. Similarly, genomic analysis of uterine lavage fluid from women without histopathological evidence of cancer, reveals the presence of driver mutations^40^. Thus, a follow-up of these patients may prove that liquid biopsy using these markers could have a predictive lead time of identifying individuals who are at risk of developing malignant tumours.

Significant numbers of cells (55%) picked were not analysable for CNA profiling due to unsuccessful amplification (QC stage). This may be due to the fact that some CTCs were undergoing necrosis or apoptosis in circulation, or possibly that haemodynamic induced anoikis may have been initiated in some cells, causing initial nuclei DNA fragmentation. In addition, this was a retrospective study where we decided to investigate CNA on previously fixed and stained CTCs mounted on slides, and thus, may not be ideal for preservation of DNA for WGA. In general, methodological improvement is needed to maximize the number of CTCs that can be genetically analysed using this method.

Overall, the number of cells analysed for these heterogenous subset of CTCs is too small and should be perceived as a hypothesis generating study. We propose that larger number of cells that are phenotypically heterogenous, should be scrutinized via molecular techniques to confirm their tumourigenic characteristics.

## Conclusion

We confirm here that combination CK/EpCAM plus CNA assessment is reliable for detection and quantification of CTCs. The lack of CNAs observed in CK/EpCAM^pos^/CD31^pos^ cells, defined here as pseudoendothelial cells, necessitates the addition of vascular endothelial markers to CTC detection panels for accurate quantification of CTCs. The presence of CNAs in the vimentin^pos^ clusters from the cystadenoma case, warrants further studies in malignant cases, as EMT-CTCs is one of the major challenges to reliably detect in HGSOC.

## Methodology

### Samples

CTCs were randomly picked from previously stained slides^18^ stored at 4 °C and also from a case with benign tumour histologically confirmed to be cystadenoma. Written consent forms were obtained from the benign cystadenoma and HGSOC patients, and all procedures were approved by the Human Research Ethics Committees at Edith Cowan University (No. 18957) and Sir Charles Gairdner Hospital (No. 2013-246 and RGS0000003289) in compliance with the Declaration of Helsinki. Experiments were performed according to institutional and national guidelines and regulations. Blood sample collection and CTC isolation were described in our previous report^18^.

### Single-cell picking, whole-genome amplification and sequencing

The CellCelector (ALS, Jena, Germany) platform was employed to pick individual cells^16^. Picked cells were individually lysed and digested, and then subjected to WGA using the Ampli1 WGA Kit (Silicon Biosystems, Bologna, Italy) according to the manufacturer’s instructions. Quality control (QC) of WGA-DNA was performed using Ampli1 QC Kit following the manufacturer’s instructions (Silicon Biosystems). Samples with > 1 PCR band were used to construct sequencing libraries. WGA-DNA from cells that passed QC were used to construct 400 bp sequencing libraries using the Ampli1 LowPass Kit for Ion Torrent (Silicon Biosystems) following the manufacturer’s instructions. Pooled library was diluted to 50 pM and loaded into an Ion 530 Chip (Life Technologies) using the Ion Chef (400 base chemistry) (Life Technologies) and sequenced on an Ion S5 (Life Technologies) for 525 flows. Reads were aligned to hg19 using Torrent Server V5.14.0 (Life Technologies). CNAs were analysed using IchorCNA (V0.2.0) using 1 Mb bins^41^. A panel of normals were constructed from white blood cells processed using the same method as described above.

### Informed consent

Informed consent was obtained from all subjects involved in the study. Both healthy volunteers and HGSOC patients signed consent forms approved by the Human Research Ethics Committees at Edith Cowan University (HREC No. 18957) and Sir Charles Gairdner Hospital (No. 2013-246 and RGS0000003289).

## Data Availability

All relevant data has been presented in the article. LP-WGS files have been uploaded in Sequence Read Archive, Reference ID: PRJNA925282. Raw images of reported results have been stored at the School of Medical and Health Sciences and at the Centre for Precision Health, Edith Cowan University. Please contact corresponding author Elin Gray, e.gray@ecu.edu.au.
